# Pre‐RAI Monocyte‐to‐Lymphocyte Ratio Predicts Early and Higher Recurrence Risk in Intermediate‐Risk DTC, While PNI and NRI Show No Prognostic Value

**DOI:** 10.1111/cen.70113

**Published:** 2026-02-23

**Authors:** Tommaso Piticchio, Francesco Galeano, Salvatore Volpe, Antonio Prinzi, Ignazio Barca, Andrea Scuto, Rosario Le Moli, Giulio Geraci, Andrea Tumminia, Sium Wolde Sellasie, Francesco Pallotti, Francesco Frasca

**Affiliations:** ^1^ Department of Medicine and Surgery University Kore of Enna Enna Italy; ^2^ Department of Clinical and Experimental Medicine, Endocrinology Section Garibaldi‐Nesima Hospital, University of Catania Catania Italy; ^3^ Department of Precision Medicine in Medical, Surgical and Critical Care (Me.Pre.C.C.) University of Palermo Italy; ^4^ Endocrine Unit, A.R.N.A.S Garibaldi‐Nesima Hospital Catania Italy; ^5^ Division of Endocrinology and Diabetes, CTO Andrea Alesini Hospital, Department of Surgical Sciences University of Rome Tor Vergata Rome Italy; ^6^ PhD School of Applied Medical‐Surgical Sciences University of Rome Tor Vergata Rome Italy

**Keywords:** differentiated thyroid cancer, monocyte‐to‐lymphocyte ratio (MLR), nutrition, recurrence, systemic inflammation

## Abstract

**Objective:**

Differentiated thyroid carcinoma (DTC) has an excellent prognosis, but recurrence remains a clinical concern, especially in intermediate‐risk patients. Current stratification systems focus primarily on tumor characteristics, overlooking the host's biological capacity to counteract tumor progression. To investigate this aspect, we assessed systemic inflammatory and nutritional status using hematological indices. The study aimed to evaluate the prognostic value of these markers in predicting recurrence risk in patients with intermediate‐risk DTC.

**Methods:**

We retrospectively analyzed consecutive patients with intermediate‐risk DTC who met the following criteria: (1) were classified as intermediate risk according to ATA guidelines; (2) showed an excellent or indeterminate response 12 months after initial treatment; and (3) had at least three consecutive years of follow‐up at our center after thyroidectomy. Nine hematological indices were calculated from blood samples collected on the day of RAI administration. Statistical analyses included ROC curve analysis, logistic regression, and Kaplan–Meier analysis.

**Results:**

A total of 282 patients were included, with a median follow‐up of 91 months. Among the indices tested, the monocyte‐to‐lymphocyte ratio (MLR) predicted recurrence better than the others. A cut‐off of 0.188 yielded 82.9% sensitivity and 54.7% specificity (AUC: 0.70). In multivariate analysis, high MLR (OR  =  4.94, *p*  <  0.001), tumor size, vascular invasion, and lymph node metastases were independently associated with recurrence. Cox regression confirmed MLR as an independent predictor of shorter recurrence‐free survival (HR  =  3.01, *p* <  0.001). Nutritional indices showed no prognostic value.

**Conclusions:**

Pre‐RAI MLR may serve as a simple marker to refine recurrence risk stratification and personalize follow‐up in intermediate‐risk DTC.

## 1 Introduction

DTC is the most common endocrine malignancy and it is characterized by a generally favorable prognosis [1, 2]. Particularly, Papillary Thyroid Carcinoma (PTC), the most common subtype, frequently presents as an indolent tumor with high overall survival (OS) rates. Despite its favorable prognosis, a non‐negligible risk of disease recurrence persists, representing a heavy psychological burden for patients with DTC and a significant challenge for clinicians in long‐term management [3, 4]. To date, major scientific societies have proposed risk stratification systems to estimate the likelihood of recurrence and guide patient management and surveillance. These systems generally agree on categorizing patients into three main risk groups: low, intermediate, and high [5, 6]. Among them, the intermediate‐risk category remains the most challenging to manage, as there is ongoing debate regarding the indication for radioactive iodine therapy (RAI) with iodine‐131 (I131) and, if indicated, whether it should be administered at ablative or adjuvant doses [4].

Importantly, these categorizations are primarily based on pathological and radiological features, implicitly assuming that recurrence risk is driven solely by tumor characteristics rather than also by the host's capacity to counteract tumor progression. However, following total thyroidectomy, the risk of recurrence may instead depend on the interplay between tumor aggressiveness and the host's defensive response. Such host‐related factors are difficult to assess and even more challenging to incorporate into daily clinical practice, where decisions need to be made quickly and based on objective criteria. This highlights the need for identifying readily accessible and reproducible biomarkers that can complement current risk stratification models and allow for a more personalized approach to post‐operative surveillance and treatment intensity. In this way, growing evidence suggests that the individual's systemic conditions, including systemic inflammation and nutritional status, play a significant role in the progression and prognosis of various cancers [7, 8, 9, 10]. Blood inflammatory biomarkers and nutritional indices could serve for the above‐mentioned scope. These indices, derived from routine complete blood count parameters, are cost‐effective, easily monitorably over time, and have already proven to be valuable tools in assessing both systemic inflammation and nutritional status, thereby providing insights beyond conventional clinical parameters [11, 12, 13, 14, 15].

However, current evidence on the use of these indices to predict recurrence risk in DTC is limited and heterogeneous. Notably, no data are currently available for the specific subgroup of intermediate‐risk DTC. Two recent meta‐analyses have reported conflicting findings on the prognostic value of the neutrophil‐to‐lymphocyte ratio (NLR) in thyroid cancer across different outcomes, including OS, progression‐free survival (PFS), disease‐free survival (DFS), and cause‐specific survival (CSS) [16, 17]. Furthermore, preliminary evidence suggests an association between preoperative platelet‐to‐lymphocyte ratio (PLR) and monocyte‐to‐lymphocyte ratio (MLR) values and adverse clinicopathological features in PTC [18, 19]. As for hematological indices reflecting nutritional status, only one study is currently available, reporting promising preliminary results regarding the role of the Prognostic Nutritional Index (PNI) in predicting lateral lymph node metastasis and recurrence‐free survival in PTC [20].

Against this background, this study aims to investigate the role of systemic inflammation and nutritional status, as assessed by hematological indices, in predicting the risk of recurrence in patients with intermediate‐risk DTC.

## 2 Materials and Methods

The study was conducted according to STrengthening the Reporting of OBservational studies in Epidemiology (STROBE) statement and its latest versions [21]. The checklist is reported in the Supporting Information Material.

### 2.1 Institutional Management of Patients with DTC

Details on the institutional management of patients with DTC are provided in another previous publication [22].

### 2.2 Inclusion and Exclusion Criteria

We included only those patients with DTC who had undergone total thyroidectomy, with or without neck lymph node dissection. Participants were required to (1) meet the criteria for intermediate recurrence risk class (an estimated risk of recurrence from 5% to 30% at baseline) according to American Thyroid Association guidelines [6]; (2) receive RAI treatment between 3 and 6 months after thyroidectomy, following levothyroxine withdrawal as described in the protocol above; (3) demonstrate either an excellent or indeterminate response to initial treatment (total thyroidectomy with RAI) at 12 months post‐surgery, based on the criteria reported by the European Society for Medical Oncology (ESMO) guidelines for thyroid cancer [5]; (4) have at least three consecutive years of follow‐up at our center following initial treatment. A full description of the histopathological inclusion and exclusion criteria is provided in our previous publication [4]. In addition, other exclusion criteria were diagnosis of diseases potentially interfering with complete blood count parameters (i.e., chronic inflammatory disease or autoimmune disease excluding Hashimoto's thyroiditis, clinical acute or chronic infections, hematologic disease, heart failure, atrial fibrillation, myeloproliferative disorders, hepatic or renal disorders, and other endocrine or metabolic disorders, including diabetes mellitus). Patients were required to demonstrate an excellent or indeterminate response to initial treatment at 12 months in order to ensure comparability in baseline recurrence risk and to avoid interference from persistent disease, which could otherwise influence systemic inflammatory indices independently of future recurrence.

### 2.3 Procedure

The study began in July 2024. We conducted a retrospective review of the digital medical records of patients consecutively referred to our institute, between 2009 and 2021, for diagnosis of DTC.

**Figure 1:**
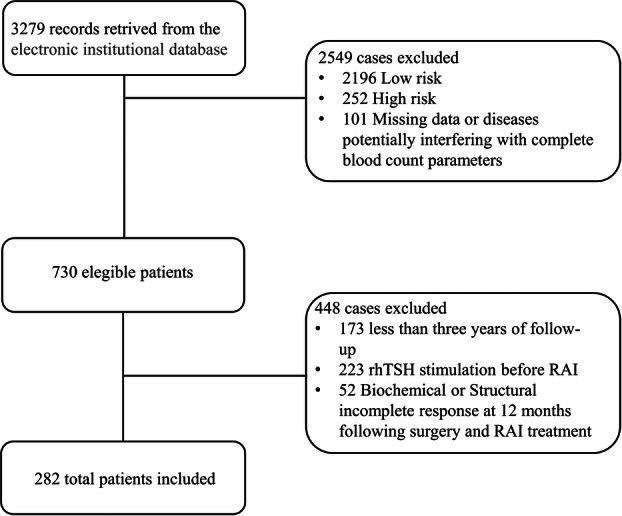
Selection flow diagram.

A total of 3279 cases were retrieved. Subsequently, we identified 730 potentially eligible patients and analyzed their medical records. A total of 282 patients were ultimately included in the study. Figure 1 illustrates the participant recruitment and selection process.

### 2.4 Systemic Inflammation and Nutritional Status

Patients' systemic inflammation and nutritional status were assessed using nine different indices calculated from the complete blood count collected on the day of RAI administration. This approach was chosen to ensure the assessment of these parameters under consistent conditions and at the same point in the disease history for each patient. Formulas for the calculation of each index are provided below:

#### 2.4.1 Inflammatory Indices

- NLR: Neutrophil (×10⁹/L)/Lymphocyte (×10⁹/L)
- PLR: Platelet (×10⁹/L)/Lymphocyte (×10⁹/L)
- MLR: Monocyte (×10⁹/L)/Lymphocyte (×10⁹/L)
- GLR (Glucose‐to‐Lymphocyte Ratio): Glucose (mg/dL)/Lymphocyte (×10⁹/L)
- SIRI (Systemic Inflammation Response Index): Neutrophil (×10⁹/L) × Monocyte (×10⁹/L)/Lymphocyte (×10⁹/L)
- AISI (Aggregate Index of Systemic Inflammation): Neutrophil (×10⁹/L) × Monocyte (×10⁹/L) × Platelet (×10⁹/L)/Lymphocyte (×10⁹/L)
- SII (Systemic Immune‐Inflammation Index): Platelet (×10⁹/L) × Neutrophil (×10⁹/L)/Lymphocyte (×10⁹/L)

#### 2.4.2 Nutritional Indices

- PNI: Albumin (g/L) + (5 × Lymphocyte (×10⁹/L))
- NRI (Nutritional Risk Index): (1.519 × Albumin [g/L]) + (41.7 × (Current weight/Ideal body weight))

Ideal body weight (IBW) was calculated using the Lorentz formula:

- Men: IBW (kg) = Height (cm) – 100 – [(Height – 150)/4]
- Women: IBW (kg) = Height (cm) – 100 – [(Height – 150)/2]

### 2.5 Clinical Follow‐Up and Definition of Recurrence

Patients were evaluated after surgery and prior to RAI administration, which was performed between 3 and 6 months after thyroidectomy. Following RAI treatment, patients were assessed after 30 days and subsequently at least once per year. During each follow‐up visit, their clinical status was assessed and classified as excellent, indeterminate, biochemical incomplete, or structural incomplete in accordance with ESMO guidelines [5], based on serum Thyroglobulin (Tg) and anti‐Thyroglobulin antibodies (TgAb) levels, as well as neck ultrasound findings. Cervical lymph nodes were classified as normal, indeterminate, or suspicious according to the criteria proposed by the European Thyroid Association (ETA) [23]. When suspicious lymph nodes were identified, fine‐needle aspiration was performed, and Tg was measured in the washout fluid to confirm structural disease. When needed, additional imaging such as CT, MRI, or FDG‐PET was used to investigate possible recurrence. Recurrence was defined as a change in the patient's response category from excellent or indeterminate to either biochemical or structural incomplete [5].

### 2.6 Participant Data

Data on the following variables were collected from the medical records of each participant: identification number, sex (male/female), age at diagnosis (years), height (cm), weight (kg), BMI (kg/m²), DTC histotype (papillary/follicular), presence of aggressive histological variants (yes/no), maximum tumor diameter (mm), multifocality (yes/no), bilaterality (yes/no), vascular invasion (yes/no), microscopic extrathyroidal extension (yes/no), lymph node metastases (yes/no), I131 dose (30/100 mCi), duration of follow‐up (months), recurrence (yes/no), and complete blood count collected the day of RAI administration, as well as Tg and TgAb. Inflammatory and nutritional indices—NLR, PLR, MLR, GLR, SIRI, AISI, SII, PNI, and NRI—were calculated as described above. The length of follow‐up was defined as the interval between initial treatment and the date of recurrence (for patients with recurrence) or the last recorded clinical evaluation (for those without).

### 2.7 Statistical Analysis

Continuous variables were expressed as medians and interquartile ranges (IQRs), while categorical variables were reported as counts and percentages. The Mann–Whitney *U* test was used to compare inflammatory and nutritional indices between patients with and without recurrence. Indices with a *p*‐value < 0.05 were selected for further analysis. Receiver operating characteristic (ROC) curve analysis was performed to assess the discriminative ability of selected indices for predicting recurrence, and the Youden index was used to determine optimal cut‐off values. Sensitivity and specificity were calculated for the most discriminative index, and the variable was dichotomized accordingly. Associations between clinical, pathological, and hematological variables and recurrence were evaluated using univariate logistic regression analysis. Variables significantly associated with recurrence (*p* < 0.05) were entered into a multivariate logistic regression model using a stepwise selection method. Model performance was assessed using McFadden's R², and collinearity diagnostics were performed by calculating variance inflation factors (VIF). Kaplan–Meier analysis was conducted to estimate recurrence‐free survival according to the categorized inflammatory index, and differences between groups were tested using the log‐rank test. Hazard ratios (HRs) and corresponding 95% confidence intervals (CIs) were calculated using Cox proportional hazards regression. The level of statistical significance was set at *p* < 0.05. All statistical analyses were performed using Jamovi (version 2.3, The Jamovi Project, 2022).

### 2.8 Ethics Statement

This research was carried out in accordance with the tenets of the 1964 Declaration of Helsinki and its later revisions. The study protocol was approved by the ethics committee of the region of Catania 2 (approval no. 12/CEL/24‐CT2). All participants provided informed consent to the use of their data in this study.

## 3 Results

A total of 282 patients were included in the study. The median age at diagnosis was 46.0 years (IQR: 36.0–55.0). Females represented the 74.1% of the cohort, with males accounting for 25.9% of cases. The median BMI was 26.6 kg/m² (IQR: 23.2–30.5), with most of the patients classified as either overweight (35.6%) or obese (29.3%), while 34.1% were of normal weight and only 1% was underweight.

Most tumors were PTC (96.8%). Aggressive variants of DTC were identified in 9.6% of patients. Specifically, tall cell (*n* = 18), diffuse sclerosing (*n* = 7), trabecular (*n* = 1), and columnar cell (*n* = 1) variants were identified. Tumor multifocality and bilaterality were observed in 36.3% and 27.7% of cases, respectively. Median maximum tumor diameter was 13.0 mm (IQR: 10.0–20.0 mm). Microscopic extrathyroidal extension was found in 50.5% of cases, and vascular invasion in 17.5%. Lymph node metastases were present in 55.8% of patients. Inflammatory indices were distributed as follows: NLR 1.88 (IQR: 1.45–2.37), PLR 114 (IQR: 93.2–141), MLR 0.184 (IQR: 0.133–0.230), GLR 40.8 (IQR: 32.1–50.6), SIRI 0.695 (IQR: 0.476–1.03), AISI 162 (IQR: 106–260), and SII 427 (IQR: 321–598). Nutritional indices were PNI with a median value of 53.8 (IQR: 50.7–57.7), while the NRI had a median value of 115 (IQR: 107–122). Two hundred forty‐seven patients received a 100 mCi dose, whereas 35 received a 30 mCi dose. The median duration of follow‐up was 94 months (IQR: 76–112). Thirteen patients had a follow‐up of 36 months, 25 ≤ 48 months, and 53 ≤ 60 months; 229 patients had a follow‐up longer than 60 months. Thirty‐five patients experienced the recurrence, all of them were treated with 100 mCi of I131. Table 1 summarizes the characteristics of the sample.

**Table 1:** Descriptive analysis of the sample included in the study.

N. of cases	282
Sex	
Female	209 (74.1%)
Male	73 (25.9%)
Age (years)	46 (36–55)
BMI (Kg/m²)	26.6 (23.2–30.5)
DTC histotype	
Papillary	272 (96.8%)
Follicular	10 (3.2%)
Aggressive histological variant	
No	255 (90.4%)
Yes	27 (9.6%)
Maximum diameter (mm)	13 (10–20)
Multifocality	
No	179 (63.7%)
Yes	102 (36.3%)
Bilaterality	
No	204 (72.3%)
Yes	78 (27.7%)
Vascular invasion	
No	227 (82.5%)
Yes	48 (17.5%)
Microscopic extra‐thyroid‐extension	
No	136 (49.5%)
Yes	139 (50.5%)
Lymph node metastases	
No	123 (44.2%)
Yes	155 (55.8%)
Dose I131	
30 mCi	35 (12.4%)
100 mCi	247 (87.6%)
Follow‐up (months)	91 (56–109)
Recurrence	
No	247 (87.6%)
Yes	35 (12.4%)
MLR	0.184 (0.133–0.230)
PLR	114 (93.2–141)
NLR	1.88 (1.45–2.37)
SIRI	0.695 (0.476–1.03)
SII	427 (321–598)
AISI	162 (106–260)
GLR	40.8 (32.1–50.6)
PNI	53.8 (50.7–57.7)
NRI	115 (107–122).

**Figure 2:**
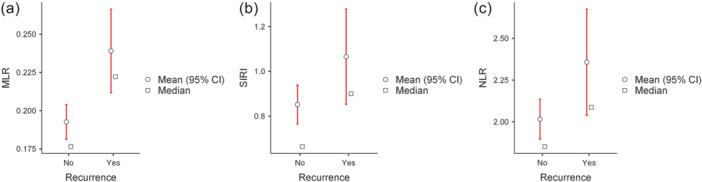
(a) Comparison of Monocyte‐to‐Lymphocyte ratio (MLR) values stratified by recurrence status. (b) Comparison of systemic inflammation response index (SIRI) values stratified by recurrence status. (c) Comparison of neutrophil‐to‐lymphocyte ratio (NLR) values stratified by recurrence status.

We performed the Mann–Whitney *U* test to assess differences in inflammatory and nutritional indices between patients with and without recurrence. MLR showed the largest difference (*p* < 0.001), followed by SIRI (*p* = 0.006) and NLR (*p* = 0.014) (Figure 2a−c).

In contrast, no significant differences were observed for PLR, GLR, AISI, SII, PNI, and NRI (all *p* > 0.05). Patients were subsequently stratified according to the time interval between surgery and RAI administration as follows: 3–< 4 months (*n* = 9), 4–< 5 months (*n* = 12), and 5–6 months (*n* = 261). A Kruskal–Wallis test was performed to compare all inflammatory and nutritional indices across the three groups. No statistically significant differences were observed for any of the evaluated indices (all H < 2.0, all *p* > 0.05). Similarly, when the time interval was dichotomized into two groups (3–< 5 months, *n* = 21 vs. 5–6 months, *n* = 261), no statistically significant differences were observed. Based on these results, we selected three indices for subsequent ROC curve and multivariate regression analyses. ROC curve analysis demonstrated that MLR had the highest discriminative ability for recurrence, with an AUC of 0.75. SIRI and NLR followed with AUCs of 0.643 and 0.628, respectively. For MLR, the optimal cut‐off identified by the Youden index was 0.188, yielding a sensitivity of 82.9% and a specificity of 54.7%. Therefore, we categorized the variable MLR according to this cut‐off (i.e., ‘Low’ ≤ 0.1875–‘High’ > 0.1875), resulting in 152 patients classified as ‘Low’ and 130 as ‘High’. The cumulative event rate was 20.9% (27/129) in the high MLR group and 5.3% (8/152) in the low MLR group.

**Figure 3:**
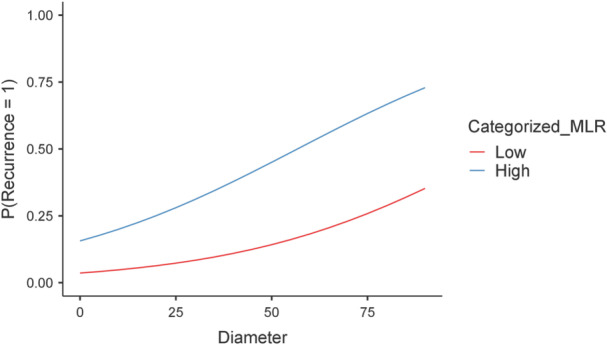
Predicted recurrence risk stratified by tumor diameter and MLR level.

Subsequently, we explored the data through the uni‐ and multi‐variate logistic regression. Univariate logistic regression analysis was performed to explore the association between clinicopathological features, inflammatory‐nutritional indices, and disease recurrence. Among the variables tested, male gender (OR = 2.81, 95% CI: 1.36–5.81, *p* = 0.005), presence of an aggressive histological variant (OR = 1.57, 95% CI: 1.15–2.62, *p*  =  0.047), maximum tumor diameter (OR = 1.03, 95% CI: 1.00–1.05, *p* = 0.04), vascular invasion (OR = 2.52, 95% CI: 1.14–5.57, *p* = 0.02), lymph node metastases (OR = 2.61, 95% CI: 1.22–5.58, *p* = 0.014), and high MLR (OR = 4.72, 95% CI: 2.10–10.81, *p* < 0.001) were significantly associated with recurrence (Table 2). These variables were included in a multivariate logistic regression analysis and the best model included high MLR (OR  =  4.94, 95% CI: 2.07–11.80, *p*  <  0.001), together with larger tumor diameter (OR = 1.03, 95% CI: 1.00–1.10, *p*  =  0.040), vascular invasion (OR = 2.61, 95% CI: 1.10–6.40, *p* = 0.035), and lymph node metastases (OR = 3.27, 95% CI: 1.30–8.25, *p* = 0.012) (Table 2) (Figure 3). The best‐fit model showed an R^2^ of 0.273. Furthermore, we detected no evidence of multicollinearity among the independent variables included in the multivariate model, with all VIF values below the conventional threshold of 5 (Table 3). Sex was not retained in the multivariate model due to a lack of incremental explanatory power after adjustment for other variables.

**Table 2:** Logistic regression analysis to evaluate factors associated with recurrence.

	Univariate analysis			Multivariate analysis		
	Odds ratio	95% CI	*p* value	Odds ratio	95% CI	*p* value
Male gender	2.81	1.36–5.81	0.005			
Age	1.02	0.99–1.04	0.265			
BMI	0.98	0.91–1.06	0.580			
Aggressive histological variant	1.57	1.15–2.62	0.047			
Multifocality	1.00	0.21–1.1	0.08			
Bilaterality	1.01	0.25–1.26	0.14			
Maximum diameter	1.03	1.0–1.05	0.04	1.03	1.00–1.10	0.040
Vascular invasion	2.52	1.14–5.57	0.02	2.61	1.1–6.40	0.035
Microscopic extra‐thyroid extension	1.2	0.583–2.42	0.636			
Lymph node metastases	2.61	1.22–5.58	0.014	3.27	1.3–8.25	0.012
NLR	1.36	0.99–1.84	0.06			
Categorized_MLR	4.72	2.1–10.81	< 0.001	4.94	2.07–11.80	< 0.001
SIRI	1.42	0.94–2.14	0.097			
PNI	0.947	0.86–1.02	0.178			
NRI	0.99	0.96–1.03	0.76			

**Table 3:** Collinearity statistics.

	VIF	Tolerance
Vascular invasion	1.05	0.956
Lymph node metastases	1.22	0.821
Categorized_MLR	1.02	0.982
Maximum diameter	1.25	0.801

**Figure 4:**
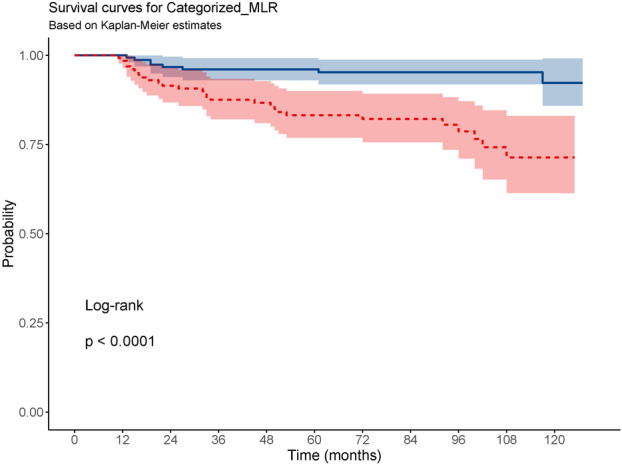
Kaplan–Meier survival curves stratified by MLR level. The continuous blue line represents the low MLR group, while the dashed red line represents the high MLR group. Shaded areas indicate 95% confidence intervals.

In Cox regression, high MLR independently affect the risk of recurrence (HR = 3.01, 95% CI: 1.71–5.27, *p* < 0.001). This finding was supported by Kaplan–Meier analysis, which showed a significantly reduced recurrence‐free survival in patients with high MLR compared to those with low MLR (log‐rank test, *p* < 0.0001) (Figure 4).

### 3.1 Head‐to‐Head Comparison of MLR With Established Biochemical Markers

**Figure 5:**
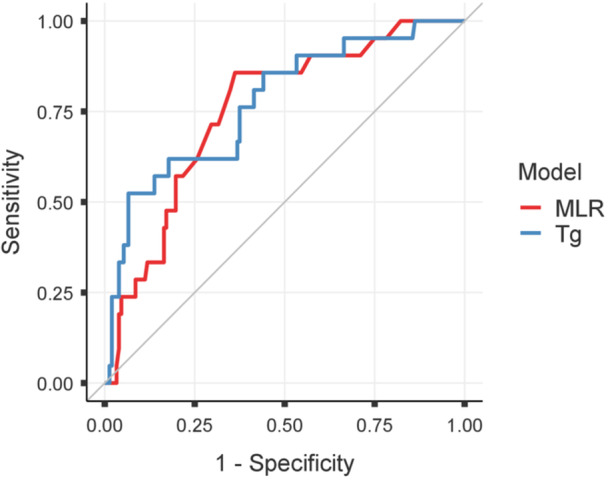
ROC curves comparing MLR and Tg. ROC curves showing the diagnostic performance of monocyte‐to‐lymphocyte ratio (MLR) and serum thyroglobulin (Tg) in predicting recurrence. The diagonal line represents the reference line for no discrimination.

**Figure 6:**
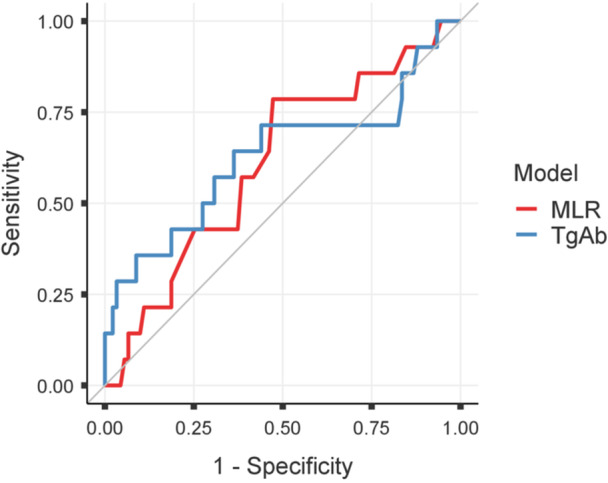
ROC curves comparing MLR and TgAb. ROC curves showing the diagnostic performance of monocyte‐to‐lymphocyte ratio (MLR) and anti‐thyroglobulin antibodies (TgAb) in predicting recurrence. The diagonal line represents the reference line for no discrimination.

We further evaluated the predictive performance of MLR through a comparison with established biochemical markers, including serum Tg in TgAb‐negative patients (*n* = 177) and TgAb in TgAb‐positive patients (*n* = 105), using ROC curve analysis. In TgAb‐negative patients, the AUC of MLR was 0.753 (95% CI 0.650–0.856), comparable to that of serum Tg (AUC 0.773, 95% CI 0.661–0.885), with no statistically significant difference between the two curves (DeLong test, *p* = 0.812) (Figure 5). In the TgAb‐positive subgroup, both MLR and TgAb showed limited discriminative ability for recurrence prediction, with an AUC of 0.611 (95% CI 0.452–0.769) for MLR and 0.630 (95% CI 0.438–0.821) for TgAb (Figure 6).

### 3.2 Exploratory Analysis According to the 2025 ATA Risk Stratification System (2025 ATA Rss)

**Figure 7:**
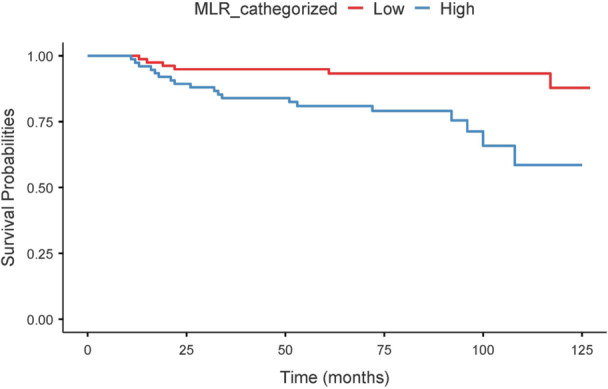
Kaplan–Meier survival curves stratified by MLR level in intermediate‐to‐high risk patients.

**Figure 8:**
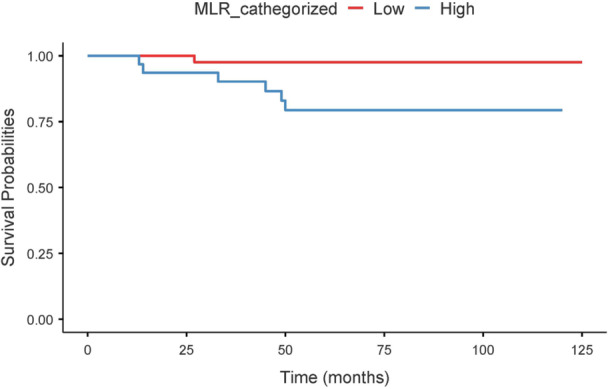
Kaplan–Meier survival curves stratified by MLR level in low‐to‐intermediate risk patients.

**Figure 9:**
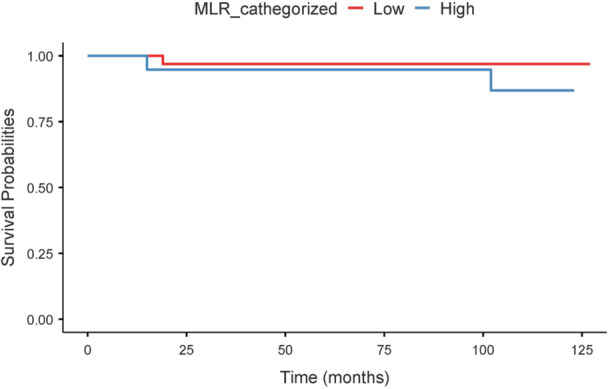
Kaplan–Meier survival curves stratified by MLR level in low‐risk patients.

It was possible to reclassify 277 patients according to the 2025 ATA rss, with the following distribution: 153 patients at intermediate–high risk, 73 at low–intermediate risk, and 51 at low risk. A total of 25 recurrence events occurred in the intermediate–high risk group, 7 in the low–intermediate risk group, and 3 in the low‐risk group. Within the 2025 ATA intermediate–high risk group, patients with high pre‐RAI MLR showed a significantly shorter recurrence‐free survival compared with those with low MLR (log‐rank *p* < 0.001). In univariable Cox regression analysis, high MLR was associated with an increased risk of recurrence (HR 2.78, 95% CI 1.44–5.38; *p* = 0.002) (Figure 7). Within the 2025 ATA low–intermediate risk group, patients with high pre‐RAI MLR exhibited a significantly shorter recurrence‐free survival compared with those with low MLR (log‐rank *p* = 0.015). Consistently, univariable Cox regression analysis showed a higher risk of recurrence in patients with high MLR (HR 4.66, 95% CI 1.04–20.84; *p* = 0.044) (Figure 8). Finally, within the 2025 ATA low‐risk group, no significant difference in recurrence‐free survival was observed between patients with high and low pre‐RAI MLR (log‐rank *p* = 0.310). Consistently, univariable Cox regression analysis did not show a significant association between high MLR and recurrence risk (HR 2.30, 95% CI 0.42–12.57; *p* = 0.337) (Figure 9).

## 4 Discussion

Although DTC typically shows excellent long‐term outcomes, patients classified as intermediate risk still experience disease recurrence in up to 30% of cases. This gap between favorable OS and appreciable recurrence rates highlights the need for further predictors available at initial staging, capable of reliably identifying patients who may benefit from closer surveillance or intensified adjuvant therapy, as well as those who may be safely reassured and appropriately managed even with a less intensive follow‐up schedule. In fact, it should be kept in mind that the risk of recurrence represents a psychological burden for patients and that long‐term serial monitoring could negatively impact their quality of life, as well as their occupational and social functioning.

In this study, we investigated whether systemic inflammatory and nutritional indices obtained after surgical recovery but before RAI administration could predict recurrence risk in patients diagnosed with intermediate‐risk DTC. Most of the available studies on the role of inflammatory indices in predicting recurrence in thyroid cancer have analyzed these values prior to surgical intervention [24]. For MLR, the optimal cut‐off identified by the Youden index was 0.188, yielding a sensitivity of 82.9% and a specificity of 54.7%. Therefore, it would be more useful to assess the inflammatory status at a standardized time point following postoperative recovery, as was done in our study.

The main finding of this study was the significant association between MLR levels and the risk of recurrence in patients with intermediate‐risk DTC, alongside a greater predictive reliability of this index compared to NLR, PLR, GLR, AISI, and SII. Specifically, patients with a pre‐RAI MLR greater than 0.188 exhibited a nearly fourfold higher recurrence rate and experienced disease recurrence significantly earlier than those with lower MLR values. The high odds and HRs reflect the narrow numerical range of MLR values, meaning small changes correspond to large relative risk increases. As a result, a hypothetical increase of a unit is theoretically associated with a very high risk. These associations persisted even after adjusting for established risk factors, including tumor size, vascular invasion, and lymph node involvement. Although the overall explanatory power of the multivariate model was modest, this is consistent with the multifactorial nature of recurrence, which is influenced by tumor biology, host genetic background and immune response, as well as environmental factors [25, 26]. In this context, MLR should be considered a complementary biomarker that adds incremental prognostic information to established clinicopathological factors rather than a standalone predictive tool. Notably, in head‐to‐head analyses, the predictive performance of MLR was comparable to that of Tg, while both MLR and TgAb showed limited discriminative ability in TgAb‐positive patients. This observation further supports the reliability of MLR in TgAb‐negative patients, whereas in TgAb‐positive individuals, it may be partly explained by the limited number of cases in this subgroup and by the presence of chronic autoimmune thyroiditis with persistent immune activation, which could affect circulating inflammatory indices and attenuate their prognostic value.

The literature on systemic inflammatory indices may be affected by potential publication bias, which should be taken into account to allow for a balanced interpretation of current evidence. Additionally, MLR is sometimes reported in the literature as the inverse ratio—namely, lymphocyte‐to‐monocyte ratio (LMR)—which limits the possibility of direct comparisons between studies. Due to its broad availability and ease calculation, the MLR has been widely assessed in various oncological research contexts. Across these studies, it has consistently demonstrated value in predicting recurrence and poorer OS or has shown significant associations with more advanced pTNM staging [27, 28, 29]. In the context of DTC, our findings are consistent with most previous studies investigating the prognostic value of MLR or LMR in patients with thyroid cancer. These studies reported significant associations between MLR and either aggressive tumor features or recurrence, although they differ substantially in terms of study design, sample size, and the timing of complete blood count collection [19, 30, 31, 32, 33]. Notably, the study most comparable to ours in terms of design, considering MLR, reported a higher risk of recurrence in patients with an MLR greater than 0.22. This cut‐off is very close to the 0.188 identified in our analysis, and the discrepancy may be attributable to the calculation of MLR within 7 days prior to surgery in that study [19]. Only one study reported no association between LMR levels and prognostic outcomes in PTC [18]. However, in that study, patients underwent a variable period of levothyroxine withdrawal ranging from 2 to 4 weeks before RAI administration. This may have influenced both the degree of hypothyroidism achieved and, consequently, the circulating levels of monocytes and lymphocytes [11]. Moreover, the enrolled patients had intermediate‐ to high‐risk PTC; thus, in a more advanced clinical setting, the impact of LMR variability may be less evident [18]. Finally, a meta‐analysis that attempted to pool log‐transformed HRs for patients with DTC found that LMR did not reach statistical significance in predicting DFS (*p* = 0.06). However, the analysis included both total thyroidectomy and hemithyroidectomy cases, did not consider the timing of data collection in relation to surgery or RAI therapy, and was limited to only nine studies published between 2018 and 2021—whereas a growing body of evidence has emerged over the last 5 years [34].

Mechanisms explaining these data are still not fully elucidated; nevertheless, hypotheses supporting the biological plausibility of our observations may be proposed on the basis of evidence already available in the literature. Monocytes and lymphocytes have been reported to play a key role in tumor immunity; accordingly, an elevated MLR may reflect a relative increase in monocytes, which can differentiate into tumor‐associated macrophages (TAMs), which have been shown to actively support tumor progression through multiple mechanisms, including promotion of angiogenesis, suppression of T‐cell function, and facilitation of metastasis [35]. Concurrently, a lower lymphocyte count suggests weakened cytotoxic T‐cell activity against residual microscopic disease. Thus, MLR serves as an integrative marker of both pro‐tumor inflammation and compromised immune surveillance. The resulting immunological disequilibrium may promote the awakening and proliferation of quiescent neoplastic cells [36]. Moreover, initial evidence supports a correlation between the concentration of immune cells within the primary tumor microenvironment and their corresponding circulating blood levels [37]. These findings provide novel insights into the potential application of MLR in clinical practice: Incorporating MLR into postoperative assessment may refine risk stratification with minimal added cost or effort, requiring a routine complete blood count before RAI. Patients with high MLR may benefit from closer follow‐up. Conversely, those with low MLR and favorable features could be candidates for de‐escalated surveillance, potentially reducing anxiety and costs.

Finally, it is also important to address the lack of significance of NLR and other neutrophil‐based indices in the regression analysis. Neutrophils primarily reflect acute inflammatory responses mediated by the innate immune system and may therefore be less representative of the chronic, tumor‐related inflammatory state. Moreover, their levels can be influenced by other transient factors, such as injury or infection, potentially limiting their prognostic reliability in this setting [38].

With regard to nutritional indices, both PNI and NRI failed to demonstrate significant prognostic value. This finding is consistent with the original purpose of these indices, which were developed for use in aggressive malignancies such as gastric, pancreatic, and hepatocellular carcinomas treated with gastrointestinal surgery [39, 40, 41], where nutritional impairment and systemic inflammation are typically more pronounced. In DTC, especially in early‐stage cases, patients typically present with preserved nutritional and immunologic profiles, limiting the utility of albumin and lymphocyte‐based indices. Supporting this, Chen et al. [42] found that PNI was only prognostically relevant in advanced DTC (TNM stage III–IV), where a threshold of preoperative PNI ≤ 53.1 independently predicted recurrence. The use of more specific nutritional markers reflecting body composition may provide greater discriminatory power in this clinical setting.

The limitations of the study should also be addressed. The main limitations are the lack of longitudinal evaluation of inflammatory and nutritional indices, which limits the ability to assess their long‐term trends, and the absence of immunohistochemical or genetic analyses in primary tumors, which restricts mechanistic insight into the findings of the present study. Although strict inclusion criteria were applied to limit confounding in inflammatory indices derived from the complete blood count, this approach may have yielded a potentially healthier and lower‐risk patient cohort. Additionally, since all patients were prepared for RAI therapy through thyroid hormone withdrawal, endogenous hypothyroidism itself may influence systemic inflammatory markers; thus, pre‐RAI MLR may reflect a composite signal of host immune status and hypothyroid‐induced inflammatory modulation. Accordingly, future studies are needed to explore the prognostic performance of MLR in unselected patients with DTC across the full spectrum of response‐to‐therapy categories, to compare cohorts prepared with recombinant human TSH, and to provide external validation before routine application in broader clinical settings.

Despite this, the study benefits from a large, highly selected cohort, with included patients starting from the same initial conditions and being uniformly followed and treated over a long‐term follow‐up, strengthening the reliability of our findings.

## 5 Conclusions

This study revealed the prognostic value of MLR levels in intermediate‐risk DTC patients. Our findings indicate that an MLR greater than 0.188, measured after surgical recovery and prior to RAI therapy, identifies patients at significantly higher and earlier risk of recurrence. Furthermore, these results are also consistent with the 2025 ATA rss. In contrast, PNI and NRI do not appear to be suitable prognostic markers for this specific patient population. The findings were instead more controversial and less clearly defined for the remaining inflammatory indices assessed. The long follow‐up period strengthens the reliability of these findings. However, further multicentric and prospective studies on larger datasets are needed to definitively validate these results.

## Author Contributions

Conceptualization: Tommaso Piticchio. Data curation: Francesco Galeano, Salvatore Volpe, Ignazio Barca, Antonio Prinzi. Formal analysis: Tommaso Piticchio, Sium Wolde Sellasie, Francesco Pallotti, Francesco Frasca. Funding acquisition; Investigation: Tommaso Piticchio. Methodology: Tommaso Piticchio, Francesco Pallotti. Project administration: Tommaso Piticchio. Resources: Francesco Frasca. Software: Tommaso Piticchio. Supervision: Francesco Frasca. Validation: Giulio Geraci, Andrea Tumminia, Rosario Le Moli, Sium Wolde Sellasie, Francesco Pallotti, Francesco Frasca. Visualization: Francesco Galeano, Salvatore Volpe, Ignazio Barca, Antonio Prinzi, Andrea Scuto, Giulio Geraci, Andrea Tumminia, Rosario Le Moli, Sium Wolde Sellasie, Francesco Pallotti, Francesco Frasca. Writing–original draft: Tommaso Piticchio. Writing–review and editing: Tommaso Piticchio, Francesco Galeano, Salvatore Volpe, Antonio Prinzi, Ignazio Barca, Andrea Scuto, Rosario Le Moli, Giulio Geraci, Andrea Tumminia, Sium Wolde Sellasie, Francesco Pallotti, Francesco Frasca.

## Funding

The authors received no specific funding for this work.

## Ethics Statement

This research was carried out in accordance with the tenets of the 1964 Declaration of Helsinki and its later revisions. The study protocol was approved by the ethics committee of the region of Catania 2 (approval no. 12/CEL/24‐CT2). All participants provided informed consent to the use of their data in this study. All procedures performed in studies involving human participants were in accordance with the ethical standards of the institutional and/or national research committee and with the 1964 Helsinki Declaration and its later amendments or comparable ethical standards. The study protocol was approved by the ethics committee of the region of Catania 2 (approval no. 12/CEL/24‐CT2).

## Consent

Informed consent was obtained from all individual participants included in the study.

## Conflicts of Interest

The authors declare no conflicts of interest.

## Supporting information

STROBE Statement—Checklist of items that should be included in reports of cohort studies.
